# Urinary [TIMP-2] × [IGFBP-7] for predicting acute kidney injury in patients undergoing orthotopic liver transplantation

**DOI:** 10.1186/s12882-019-1456-1

**Published:** 2019-07-17

**Authors:** Judith Schiefer, Paul Lichtenegger, Gabriela A. Berlakovich, Walter Plöchl, Claus G. Krenn, David M. Baron, Joanna Baron-Stefaniak, Peter Faybik

**Affiliations:** 1 Department of Anaesthesia, Intensive Care Medicine and Pain Medicine, Medical University of Vienna, Waehringer Guertel 18-20, 1090 Vienna, Austria; 2 Department of Surgery, Division of Transplantation, Medical University of Vienna, Waehringer Guertel 18-20, 1090 Vienna, Austria

**Keywords:** Acute kidney injury, Liver transplantation, Urinary biomarker, TIMP-2, IGFBP-7

## Abstract

**Background:**

The product of the concentrations of urinary tissue inhibitor of metalloproteinases-2 and insulin-like growth factor binding protein-7 (urinary [TIMP-2] × [IGFBP-7]) has been suggested as biomarker for early detection of acute kidney injury (AKI) in various clinical settings. However, the performance of urinary [TIMP-2] × [IGFBP-7] to predict AKI has never been assessed in patients undergoing orthotopic liver transplantation (OLT). Thus, the aim of this study was to assess the early predictive value of urinary [TIMP-2] × [IGFBP-7] for the development of AKI after OLT.

**Methods:**

In this observational study, urinary [TIMP-2] × [IGFBP-7] was measured in samples from adult OLT patients. AKI was diagnosed and classified according to KDIGO criteria. Areas under the receiver operating curves (AUC) were calculated to assess predictive values of urinary [TIMP-2] × [IGFBP-7] for the development of AKI.

**Results:**

Forty patients (mean age 55 ± 8 years) were included. Twenty-eight patients (70%) developed AKI stage 1, 2, or 3 within 48 h after OLT. Urinary [TIMP-2] × [IGFBP-7] was not predictive for AKI at the end of OLT (AUC: 0.54, CI [0.32–0.75], *P* = 0.72), at day 1 (AUC: 0.60, CI [0.41–0.79], *P* = 0.31), or day 2 after OLT (AUC: 0.63, CI [0.46–0.8], *P* = 0.18).

**Conclusion:**

Based on our results, routine clinical use of urinary [TIMP-2] × [IGFBP-7] cannot be recommended for risk assessment of AKI in patients undergoing OLT.

## Background

Acute kidney injury (AKI) is a frequent complication after orthotropic liver transplantation (OLT) and is associated with increased morbidity and mortality, poor graft survival, and prolonged hospital length-of-stay [1–3]. According to previous studies, more than 50% of patients undergoing OLT develop AKI [1, 4–7], and roughly 30% of these patients require postoperative renal replacement therapy (RRT) [3, 7, 8]. Early institution of renal replacement therapy (RRT) in patients with severe AKI may improve survival [9, 10]. Therefore, early recognition of postoperative kidney dysfunction is essential in order to optimize outcome.

In daily clinical practice, changes in serum creatinine (sCr) concentrations and urine output are used to evaluate and diagnose AKI [11]. However, these “traditional” kidney parameters have limitations related to early and accurate identification of AKI [12]. Furthermore, sCr has limitations when used as a marker to diagnose AKI in patients with end-stage liver disease (ESLD) undergoing OLT. Patients with ESLD frequently show altered biosynthesis of creatinine, a reduction in muscle mass, malnutrition, reduced protein and creatine intake, as well as increased concentrations of serum bilirubin [12, 13]. Creatinine assays using Jaffe’s reaction may overestimate renal function due to interference by non-creatinine chromogens such a bilirubin [13]. In addition, early detection of AKI using sCr concentrations is limited by the fact that sCr concentrations increase when renal function has already deteriorated [14]. Furthermore, the need for massive fluid or blood products transfusion during OLT resulting in fluid overload can mask the increase in sCr, delaying diagnosis of AKI. Novel biomarkers might facilitate early diagnosis of AKI after OLT, making identification of these biomarkers a major clinical interest.

Cell cycle arrest proteins have been suggested as early indicators of AKI [15, 16]. In particular, urinary tissue inhibitor of metalloproteinases-2 (TIMP-2) and insulin-like growth factor binding protein-7 (IGFBP-7) are biomarkers of the G1 renal tubular cell cycle arrest at the early phase of AKI. The product of the urinary concentrations of TIMP-2 and IGFBP-7 (urinary [TIMP-2] × [IGFBP-7]) has been shown to be a promising biomarker for early prediction of AKI in various clinical settings such as out-of-hospital cardiac arrest, in critically ill patients, and following major surgery or emergency department admission [17–22]. As the predictive value of urinary [TIMP-2] × [IGFBP-7] for the development of AKI has never been assessed in patients undergoing OLT, we hypothesized urinary [TIMP-2] × [IGFBP-7] can predict AKI after OLT. Therefore, the aim of the present study was to determine whether urinary [TIMP-2] × [IGFBP-7] in the early perioperative phase of OLT, and assess the performance of urinary [TIMP-2] × [IGFBP-7] in predicting the development of AKI.

## Methods

### Study subjects and classification of AKI

This observational study was performed at the General Hospital of Vienna of the Medical University of Vienna in accordance with the ethical standards laid down in the Declaration of Helsinki and the Declaration of Istanbul. Prior to commencement of the study, we obtained institutional ethics committee approval (reference number 1148/2014). Patients with ESLD undergoing OLT at the Medical University of Vienna between September 2014 and December 2015 were screened for enrollment. Exclusion criteria were preoperative RRT, veno-venous bypass during OLT, combined liver-kidney transplantation, the need for surgical revision within 48 h after OLT, and high-urgency liver transplantation. Prior to inclusion, written informed consent was signed by all patients.

### Anesthesia, surgery, and immunosuppression

All OLTs were performed under general anesthesia using the local standard technique with bi-caval clamping without veno-venous bypass. Surgical techniques including partial clamping of the caval vein combined with temporary porto-caval shunting and piggy-back or side-to-side cavo-cavostomy are not routinely performed at our center. Patients were routinely admitted to the intensive care unit after surgery, and immunosuppression was administered according to the local standardized protocol for immunosuppression in OLT, as previously described [23].

### Data collection and acute kidney injury

Demographic and epidemiologic data were collected before surgery. In order to assess the severity of liver disease we calculated the model for end-stage liver disease (MELD) score. Furthermore, perioperative parameters including laboratory values, duration of surgery, cold ischemia and caval clamping as well as blood loss, transfusion of packed red blood cells, platelets and fresh frozen plasma, fluid balance and urine output was recorded using the local patient data management system (Phillips Healthcare, Hamburg, GER).

The KDIGO criteria were applied to diagnose and classify postoperative AKI [11]. Serum creatinine and urine output were examined daily to define the AKI stage. Acute kidney injury was diagnosed when KDIGO criteria were met within 48 h after OLT. Patients not developing AKI (no AKI group) were compared to patients who developed any stage of AKI (AKI group) and to those who developed AKI stage 2 and 3 (severe AKI).

### Sample collection and processing

Urine sample collection included following time points: after induction of anesthesia and placement of the urinary catheter (baseline, T0); at the end of OLT (T1); on the first postoperative day, 24 h after reperfusion (T2); and on the second postoperative day, 48 h after reperfusion (T3). Following collection, urinary [TIMP-2] × [IGFBP-7] was measured using the Nephrocheck® test (Astute Medical, Paris, FRA) according to the manufacturer’s protocol. Urinary [TIMP-2] × [IGFBP-7] values are displayed as (ng/ml)^2^/1000.

### Statistical analysis

Statistical analysis was performed using Prism 6.0 (GraphPad Software, La Jolla, CA). Data are expressed as mean ± standard deviation. A Kolmogorov-Smirnov test was used to verify normality of measured values. Data were analyzed using ANOVA or Friedman’s test with multiple comparisons for differences within groups, and with t-test or Whitney test for differences among two groups. A receiver operating characteristics (ROC) curve analysis was performed for urinary [TIMP-2] × [IGFBP-7] as predictor of any AKI (AKI stage 1 + 2 + 3) and as predictor of severe AKI (i.e. AKI stage 2 and 3). Furthermore, we performed regression analysis including clinically significant covariates known to be associated with AKI after OLT in order to assess predictive significance of urinary [TIMP-2] × [IGFBP-7]. An adjusted *P*-value < 0.05 was considered significant for all statistical analyses.

Sample size calculation was performed using MedCalc (MedCalc Software bvba, Ostend, Belgium), yielding an estimated sample size of 38 patients. The calculation was performed with type I error of 0.05, type II error of 0.2 (power 80%), the AUC expected to be found in the study of 0.70, the null hypothesis AUC of 0.5, and ratio of sample sizes in negative/positive groups (patiens with AKI stage 0 + 1/AKI stage 2 + 3 = 1), according to previous studies performed in our transplant center [7, 24].

## Results

### Preoperative patients’ characteristics and incidence of AKI

Forty-six consecutive patients were screened for enrollement. Six patients were excluded (1 due to combined OLT with kidney transplantation, 1 patient was on intermittend renal replacement therapy prior to OLT, 2 patients due to high urgency OLT, 2 patients due to need for surgical revision within 48 h). Other than a higher MELD score due to increased sCr and need of preoperative RRT, the characteristics of the excluded patients did not differ from the rest of study population (data not shown). Forty patients with end-stage liver disease were included in our study. Table 1 depicts demographic characteristics of the study population. Within 48 h after OLT, 15 patients (37%) developed stage 1 AKI, 9 patients (23%) developed stage 2 AKI, and 4 patients (10%) developed stage 3 AKI, while 12 patients (30%) did not develop AKI. Four patients required renal replacement therapy during the first week of the ICU stay. Preoperative sCr concentrations, glomerular filtration rate, the MELD score and body-mass index, as well as intraoperative transfusion requirements, cold ischemia time and caval clamping time did not differ between the no AKI group and the AKI group (Table 2). The mean blood lactate at the time of admission to ICU was 2.6 + 2.1 mmol/l, and not differ among patient with AKI and those who did not develop AKI.

**Table 1:** Demographic data of the study population

Sex	
Male, n (%)	29 (73)
Female, n (%)	11 (27)
Ethnicity	caucasian
Etiology of end-stage liver disease	
Alcohol-induced, n (%)	18 (45)
Viral, n (%)	8 (20)
Hepatocellular carcinoma, n (%)	4 (10)
Autoimmune, n (%)	4 (10)
Primary biliary cirrhosis, n (%)	2 (5)
Other, n (%)	4 (10)
Categorical variables are described by absolute and relative frequencies	

Results Section: Preoperative patients’ characteristics and incidence of AKI (p.8)

**Table 2:** Perioperative characteristics of patients undergoing OLT

	All patients	No AKI	AKI	*P* value
Patients (n)	40	12	28	
Age (years)	56 ± 8	58 ± 8	56 ± 10	0.53
MELD	17 ± 6	17 ± 8	17 ± 9	0.98
eGFR (mL/min/1.73 m^2^)	86 ± 31	85 ± 34	89 ± 29	0.37
Preoperative sCr (mg/dl)	0.9 ± 0.4	1.1 ± 0.5	0.9 ± 0.3	0.09
Body-mass index	27 ± 5	27 ± 4	27 ± 5	0.84
Cold ischemia time (min)	340 ± 145	351 ± 182	332 ± 60	0.67
Caval clamping time (min)	82 ± 16	83 ± 26	84 ± 16	0.97
PRBC units transfused, n	4 ± 3	4 ± 3	4 ± 4	0.83
FFP units transfused, n	6 ± 6	7 ± 7	6 ± 6	0.48
Thrombocyte units transfused, n	1 ± 1	1 ± 1	1 ± 0	0.80

Data are depicted as mean ± standard deviation. *P* values indicate differences among the no AKI and AKI group

Abbreviations: *AKI* acute kidney injury, *eGFR* estimated glomerular filtration rate, *FFP* fresh frozen plasma, *MELD* model for end-stage liver disease, *PRBC* packed red blood cell, *sCr* serum creatinine

Results Section: Preoperative patients’ characteristics and incidence of AKI (p.8)

### Urinary [TIMP-2] × [IGFBP-7] in patients undergoing OLT

**Fig. 1:**
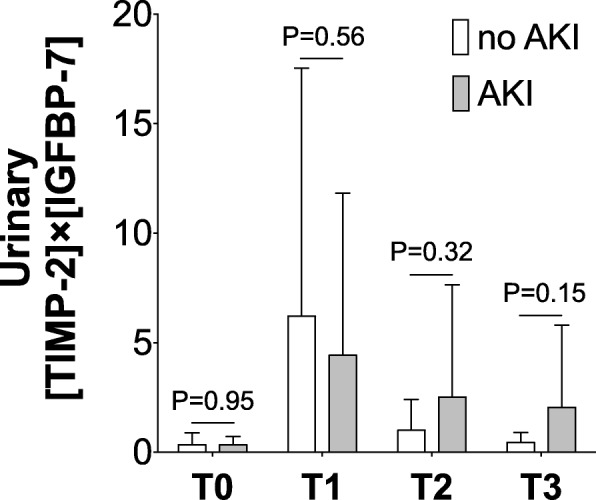
Urinary [TIMP-2] × [IGFBP-7] at following time points: T0 (baseline, under anesthesia before skin incision), T1 (at the end of surgery), T2 (24 h after graft reperfusion on day 1 after OLT), and T3 (48 h after reperfusion on day 2 after OLT). White bars indicate values of patients without AKI, gray bars represent values of patients who developed stage 1, 2 or 3 AKI after undergoing OLT. *P* values indicate differences among groups

Baseline urinary [TIMP-2] × [IGFBP-7] was 0.34 ± 0.55 in patients without AKI and 0.34 ± 0.39 in patients who developed AKI after OLT (*P* = 0.95, Fig. 1). At the end of OLT (T1), urinary [TIMP-2] × [IGFBP-7] increased to 6.22 ± 11.32 in the no AKI group (*P* = 0.02 versus baseline) and to 4.43 ± 7.39 in the AKI group (*P* = 0.009 versus baseline). At day 1 after OLT (T2), urinary [TIMP-2] × [IGFBP-7] did not differ from baseline in patients without AKI (1.00 ± 1.41, *P* = 0.98) or those with AKI (2.52 ± 5.13, *P* = 0.29). Similarly, at day 2 after OLT (T3), urinary [TIMP-2] × [IGFBP-7] did not differ from baseline in the no AKI group (0.45 ± 0.44, *P* = 0.99) or in the AKI group (2.06 ± 3.76, *P* = 0.50). Urinary [TIMP-2] × [IGFBP-7] did not differ among patients without AKI and those with AKI at any time point measured (Fig. 1).

### Predictive value for AKI after OLT

In order to evaluate the predictive value of urinary [TIMP-2] × [IGFBP-7] for the development of AKI within 48 h after OLT, ROC curve analyses were performed. At the end of OLT (T1), at day 1 (T2) and day 2 after OLT (T3) urinary [TIMP-2] × [IGFBP-7] did not predict the development of AKI stage 1, 2 and 3 after OLT (Table 3). In addition, our results revealed that urinary [TIMP-2] × [IGFBP-7] had no power to predict the development of moderate to severe AKI (AKI stage 2 + 3) after OLT at any of the time points measured (Table 3).

**Table 3:** Area under the ROC for development of any AKI and severe AKI

	AUC	95% CI	*P* value
Areas under the ROC curve for the development of any stage of AKI			
T1	0.54	0.32–0.75	0.72
T2	0.60	0.41–0.79	0.31
T3	0.63	0.46–0.8	0.18
Areas under the ROC curve for the development of severe AKI			
T1	0.51	0.26–0.75	0.96
T2	0.64	0.42–0.87	0.21
T3	0.71	0.50–0.92	0.08

Abbreviations: *AKI* acute kidney injury, *ROC* receiver operating curve, *CI* confidence interval, *T* time point

Results section: Predictive value for AKI after OLT (p.10)

As KDIGO criteria use a time frame of 7 days to diagnose AKI, data were further analyzed using this time frame. Even when extending the period for diagnosis of AKI to 7 days, urinary [TIMP-2] × [IGFBP-7] did not differ among patients without AKI and those with AKI (data not shown).

In regression analysis including the covariates age, diabetes, amount of transfused units of red blood cells, fresh frozen plasma and platelets, caval clamping time and baseline creatinine, urinary [TIMP-2] × [IGFBP-7] measured at T1, T2 and T3 was not associated with any AKI (*P* = 0.09, *P* = 0.54 and *P* = 0.15, respectively) and severe AKI (P = 0.1, *P* = 0.77 and *P* = 0.82, respectively). In patients who developed severe AKI, only baseline creatinine remained in the multivariate analysis model (*P* = 0.006).

## Discussion

In the current study, we measured urinary [TIMP-2] × [IGFBP-7] in patients undergoing OLT and assessed its ability to predict the development of AKI after OLT. Urinary [TIMP-2] × [IGFBP-7] increased significantly in patients following OLT, but did not differ among patients who developed AKI and those with normal postoperative kidney function. In our patients undergoing OLT, urinary [TIMP-2] × [IGFBP-7] was not predictive for the development of AKI.

Several studies have reported that urinary [TIMP-2] × [IGFBP-7] predicts the development of AKI after major surgery [18, 19, 21], emergency department admission [25], out-of-hospital cardiac arrest [20], and in critically ill patients [17, 26]. In critically ill patients, the urinary [TIMP-2] × [IGFBP-7] was suggested as a better predictor for AKI than urinary kidney injury molecule 1, plasma cystatin C, and urinary and plasma neutrophil gelatinase–associated lipocalin (NGAL) [17]. After several promising studies, commercially available devices for bed-side assessment of urinary [TIMP-2] × [IGFBP-7] became available, and received clearance by the US food and drug administration (FDA) for clinical use as an aid, but not as a stand-alone test, in risk assessment of AKI. As a result, more and more studies assessing the performance of urinary [TIMP-2] × [IGFBP-7] in predicting the development of AKI are being published, partially yielding controversial results.

In particular, controversial data have been reported regarding the performance of urinary [TIMP-2] × [IGFBP-7] in predicting AKI in patients undergoing major cardiac and non-cardiac surgery. While some investigators demonstrated that urinary [TIMP-2] × [IGFBP-7] was a sensitive and specific early biomarker in identifying AKI following cardiac surgery [18, 21], Wetz et al. reported that urinary [TIMP-2] × [IGFBP-7] could not discriminate patients with a KDIGO score of 0 from those with a KDIGO score of 1 or 2 [27]. Furthermore, Finge et al. found that urinary [TIMP-2] × [IGFBP-7] could not accurately predict the occurrence of postoperative AKI in patients undergoing cardiac surgery with cardiopulmonary bypass [28]. It is worth mentioning that cell cycle arrest markers are influenced by pulmonary, cardiovascular or metabolic comorbidities [29]. Thus, these contradicting results between studies might be explained by inclusion of patients with or without certain comorbidities.

With respect to non-cardiac surgery, Gocze et al. proposed that urinary [TIMP-2] × [IGFBP-7] predicts the development of AKI in patients following major non-cardiac surgery. However, when performing subgroup analysis only assessing patients undergoing hepatic surgery, the authors reported greater urinary [TIMP-2] × [IGFBP-7] which was not associated with the development of AKI. The authors explained these conflicting findings with early postoperative correction of the perioperative fluid depletion, which might have resolved cell cycle arrest and prevented the development of AKI [19]. Our results support the findings of Gocze et al., as we observed an increase of urinary [TIMP-2] × [IGFBP-7] in all patients following OLT, but were not able to show a predictive value of urinary [TIMP-2] × [IGFBP-7] for the development of AKI.

Studies indicating a poor performance of urinary [TIMP-2] × [IGFBP-7] as an early predictor for AKI suggest that urinary [TIMP-2] × [IGFBP-7] is of limited clinical value for early diagnosis of AKI, at least in patients following major surgery. Our findings propose that urinary [TIMP-2] × [IGFBP-7] is not useful as a diagnostic aid for early detection of AKI in patients undergoing OLT. Based on these data, it remains questionable whether bed-side assessment of urinary [TIMP-2] × [IGFBP-7] should be recommended and routinely preformed in daily clinical practice in postoperative patients. However, several large clinical studies have successfully demonstrated that urinary [TIMP-2] × [IGFBP-7] identified critically ill patients at risk for imminent AKI [17, 30–32]. Of note, clinical use of bed-side assessment devices was accredited for clinical evaluation of patients admitted to the ICU with acute cardiovascular or respiratory compromise. Therefore, further studies are required to prove the performance urinary [TIMP-2] × [IGFBP-7] as an early predictor for AKI in the postoperative setting.

This study has several limitations. We may fail to detect a signal due to the small sample size of only 40 OLT patients. Biomarker studies normally use hundreds of patients to get an accurate reading on the test performance. Thus, the study could be underpowered to exclude clinically relevant performance. However, the incidence of AKI in our population of OLT patients is rather high with 70%, and based on the sample size calculations from our previous data, there shoud be a signal if the test would be sensitive in this specific population.

Furthermore, the small sample size in our study did not permit to reliably analyze whether other important risk factors such as MELD score, intraoperative blood loss, blood transfusion or fluid administration affected the performance of urinary [TIMP-2] × [IGFBP-7] in predicting AKI after OLT. The fact that many known risk factors for AKI in cirrhosis patients did not differ among patients with and without AKI, suggests that their potentially deleterious effects were to weak to be detected in our patient population. We were also unable to analyze whether the presence of comorbidities affected the performance of urinary [TIMP-2] × [IGFBP-7] to predict AKI after OLT. In our study population, mean MELD score was rather low, so we cannot conclude on more severely ill liver transplant patients, who might even have a higher risk of AKI. Therefore, the results of our study cannot be extrapolated to patients with greater severity of disease. In addition, we also included patients with a preoperative estimated glomerular filtration rate (eGFR) below 60 mL/min/1.73 m^2^, which would suggest impaired preoperative kidney function. However, due to the low patient number with impaired preoperative kidney function, we did not perform sub-analyses in this patient population. Thus, the predictive value of urinary [TIMP-2] × [IGFBP-7] might be different in patients with or without pre-existing kidney impairment. Nevertheless, a clinically useful biomarker should predict AKI regardless of the patients’ comorbidities and the pre-existing kidney impairment.

## Conclusion

In this study, urinary [TIMP-2] × [IGFBP-7] did not predict the development of AKI in patients undergoing OLT. Our findings suggest that urinary [TIMP-2] × [IGFBP-7] might not be a promising diagnostic tool for early detection of AKI in patients undergoing OLT.

## Data Availability

The datasets used and analyzed during the current study are available from the corresponding author upon reasonable request.

## Abbreviations

AKI: Acute kidney injury
AUC: Area under the receiver operating curve
eGFR: Estimated glomerular filtration rate
FFP: Fresh frozen plasma
IGFBP-7: Insulin-like growth factor binding protein-7
MELD: Model for end-stage liver disease
OLT: Orthotopic liver transplantation
PRBC: Packed red blood cells
sCr: Serum Creatinine
T: Time point
TIMP-2: Tissue inhibitor of metalloproteinases-2
